# Isothiocyanates induce oxidative stress and suppress the metastasis potential of human non-small cell lung cancer cells

**DOI:** 10.1186/1471-2407-10-269

**Published:** 2010-06-09

**Authors:** Xiang Wu, Yu Zhu, Huiqin Yan, Boning Liu, Ying Li, Qinghua Zhou, Ke Xu

**Affiliations:** 1 Tianjin Key Laboratory of Lung Cancer Metastasis and Tumor Microenviroment, Tianjin Lung Cancer Institute, Tianjin Medical University General Hospital, Tianjin 300052, China

## Abstract

**Background:**

Isothiocyanates are natural compounds found in consumable cruciferous vegetables. They have been shown to inhibit chemical carcinogenesis by a wide variety of chemical carcinogens in animal models. Recent studies have also shown that isothiocyanates have antitumor activity, inhibiting the growth of several types of cultured human cancer cells. Our previous study showed that PEITC inhibited human leukemia cells growth by inducing apoptosis. However, the effect of isothiocyanates on lung cancer cell metastasis has not been studied. In the present study, we investigated the inhibitory effects of BITC and PEITC on metastatic potential of highly metastatic human lung cancer L9981 cells.

**Methods:**

Cell migration and invasion were measured by wound healing assay and transwell chemotaxis assay. Expression of metastasis-related genes was assessed by quantitative RT-PCR and Western blotting. The mechanisms of action were evaluated by flow cytometry, reporter assay and Western blotting.

**Results:**

Our data showed that both BITC and PEITC inhibited L9981 cell growth in a dose-dependent manner, the IC50 values were 5.0 and 9.7 μM, respectively. Cell migrations were reduced to 8.1% and 16.5% of control, respectively; and cell invasions were reduced to 2.7% and 7.3% of control, respectively. Metastasis-related genes MMP-2, Twist and β-catenin were also modulated. BITC and PEITC inhibited cell survival signaling molecules Akt and NFκB activation. Moreover, BITC and PEITC increased ROS generation and caused GSH depletion. Pretreatment with NAC blocked BITC and PEITC induced ROS elevation and NFκB inhibition.

**Conclusion:**

Our results indicated that BITC and PEITC suppress lung cancer cell metastasis potential by modulation of metastasis-related gene expression, inhibition of Akt/NFκB pathway. Induction of oxidative stress may play an important role.

## Background

Lung cancer is the most common cancer worldwide, with approximately 1.3 million cases recorded annually [1]. Furthermore, lung cancer is the leading cause of cancer-related deaths and is responsible for 1.18 million deaths annually [2]. The 5-year relative survival rate is approximately 15% [3]. Most patients present with locally advanced (37%) or metastatic (38%) disease at the time of diagnosis [4]. Surgery, chemotherapy and radiation have been generally unsatisfactory, especially in the treatment of advanced disease. As 90% of lung cancer patients die of metastasis [5], metastasis is of great importance to the clinical management.

Metastasis is an extraordinarily complex process, several discrete steps are discernable in the biological cascade of metastasis: loss of cellular adhesion, increased motility and invasiveness, entry and survival in the circulation, exit into new tissue, and eventual colonization of a distant site [6]. A wide variety of factors contributing to the spread of tumor cells includes cytokines, hormones, growth factors, cell adhesion molecules, and extracellular matrix proteins [7]. To date, the mechanism of metastasis is unclear, new strategies based on better understanding of the mechanism are clearly needed to improve the treatment efficacy of this fatal disease.

Numerous studies support the fact that phytochemicals found in certain food substances protect against cancer. Cruciferous vegetables have been widely accepted as potential diet components that may decrease the risk of cancer [8]. Isothiocyanates are abundant in cruciferous vegetables such as broccoli, watercress, Brussels sprouts, cabbage, Japanese radish and cauliflower, they play a significant role in cancer chemopreventive activity of these vegetables. Some isothiocyanates derived from cruciferous vegetables, such as phenethyl isothiocyanate (PEITC), benzyl isothiocyanat (BITC), sulforaphane (SFN) are highly effective in preventing or reducing the risk of cancer induced by carcinogens in animal models [9]. The mechanisms of cancer chemopreventive activity of isothiocyanates are the inhibition of phase I enzymes cytochrome P-450s involved in the activation of carcinogen and/or induction of phase II detoxifying enzymes, such as glutathione S-transferases, quinone reductase, and UDP-glucuronosyltransferases [9]. The induction of phase II enzymes is mediated by Nrf2-dependent pathway [10]. Recent studies have also shown that isothiocyanates have antitumor activity, inhibiting the growth of several types of cultured human cancer cells. Isothiocyanates induce cancer cell apoptosis [11], cell cycle arrest [12], generation of reactive oxygen species (ROS) [12,13], regulate the activation of transcription factors STAT3, NFκB and Nrf2 [10,14,15], inhibit MAPK and PKC activities [12,16], down-regulate estrogen receptor [17] etc. However, the mechanism is not fully understood.

**Figure 1:**
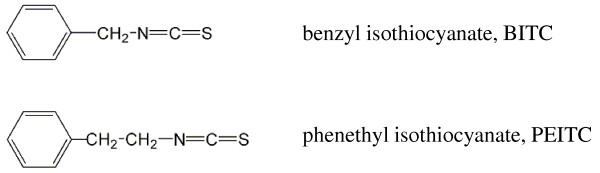
**Structures of BITC and PEITC**.

In this study, we focused on two isothiocyanates: BITC and PEITC (Figure 1), investigated their inhibitory activities on lung cancer cell metastasis potential. We have established a pair of highly metastatic human large cell lung cancer cell line L9981 and low metastatic cell line NL9980, and examined the effect of BITC and PEITC on cell proliferation, invasion, migration, and expression of metastasis-related genes.

## Methods

### Materials

PEITC, BITC, NAC were purchased from Sigma Chemical Co. (St. Louis, MO). Rabbit monoclonal antibodies against Twist, MMP-2, polyclonal antibodies against Akt, p-Akt were purchased from Cell Signaling (Beverly, MA), mouse monoclonal antibody against β-actin were purchased from SIGMA, secondary antibodies coupled to HRP were purchased from ZSGB-BIO (Beijing, China). Trizol was purchased from Invitrogen (Carlsbad, CA), reverse transcription kit and real-time PCR kit were purchased from TaKaRa Biotechnology Co. (Dalian, China). pNFκB-luc was purchased from Clontech (Mountain View, CA), pRL-SV40 was purchased from Promega (Madison, WI).

### Cell lines

Highly metastatic cell line L9981 and low metastatic cell line NL9980 were established from a human lung large cell carcinoma cell line (WCQH29801) [18]. Cells were grown and maintained in RPMI-1640 medium supplemented with 10% fetal bovine serum, 2 mmol/L glutamine (GIBCO BRL, Grand Island, NY) at 37°C, 5% CO_2_. Penicillin and streptomycin were not added into culture medium to avoid the cross effects with isothiocyanates.

### Cell proliferation assay

Cells were seeded at an initial density of 2 × 10^5 ^cell/mL and incubated with 1 - 40 μM PEITC or BITC for 48 h at 37°C. Stock solutions of the compounds (100 mM) were prepared in DMSO and diluted into the growth medium such that the final concentration of DMSO did not exceed 0.05% (v/v), a concentration that did not induce toxicity in L9981 or NL9980 cells. The cell viability were determined by Vi-CELL Cell Viability Analyzer (BECKMAN COULTER, Brea, CA), following the manufacture's instruction. The median inhibitory concentration IC_50 _values were calculated using GraphPad Prism 5.0 software (La Jolla, CA).

### Would healing assay

Cell migration was examined using a wound healing assay. Cells were cultured in six-well plates to 100% confluence. A plastic pipette tip was used to generate a clean wound area across the center of the well. Cell debris was removed by washing with PBS, and cells were allowed to migrate in the medium. The wound was assessed by a microscope (Nikon, Tokyo, Japan) at ×40 magnification at indicated time points. Cells in each field of view were counted by photographing through the microscope, and the average number of cells present in each scrape with each treatment was determined. At least five wound areas were investigated on each plate to quantify the migration.

### Cell invasion assay

The tumor cell invasion activity was assessed by Cell Invasion Kit (CHEMICON INTERNATIONAL Inc., Billerica, MA). It was performed in an Invasion Chamber, a 24-well tissue culture plate with cell culture inserts. The inserts contain an 8 μm pore size polycarbonate membrane, over which a thin layer of ECMatrixTM is dried. L9981 cells were suspended to a final concentration of 2 × 10^5 ^cell/mL in serum free medium with 0.1% BSA. Cell suspensions (300 μL) were added to the upper compartment, medium collected from NIH3T3 cell culture was added with 0.1% BSA, then added to the lower compartment, and incubated for 24 h at 37°C in 5% CO_2 _atmosphere. Invasive cells on lower surface of the membrane were stained following the manufacturer's instruction, and counted by photographing the membrane through the microscope (×200 magnification).

### Western blotting analysis

Western blottings were performed as previously described [19]. Briefly, L9981 cells were incubated with PEITC or BITC for 24 h, washed with PBS, and the cell pellets were lysed in lysis buffer (20 mM Tris (pH7.5), 150 mM NaCl, 1% Triton X-100, sodium pyrophosphate, β-glycerophosphate, EDTA, Na_3_VO_4_, leupeptin) (Beyotime Institute of Biotech, Jiangsu, China) for 30 min on ice. Lysates were centrifuged (15,000 g, 20 min, 4°C). Five-fold concentrated SDS sample buffer (Beyotime Institute of Biotech, Jiangsu, China) was added to cell lysates, boiled for 5 min, and electrophoresed on a 12% SDS-polyacrylamide gel. Protein molecular weight standards (Bio-Rad, Richmond, CA) were run concurrently. Proteins were transferred electrophoretically to nitrocellulose membranes. Membranes were blocked for 1 hour at room temperature with 5% milk protein, 0.1% Tween 20 in PBS (PBS-Tween), then were probed with rabbit anti-Twist, MMP-2, Akt, p-Akt antibodies at 1:1000 dilution in PBS-Tween with 5% BSA overnight at 4°C. After washing, membranes were probed with HRP-conjugated goat anti-rabbit antibody at 1:5000 dilution in PBS-Tween with 3% milk protein for 1 hour. After washing, blots were developed with the Phototope HRP Western Blot Detection system (Cell Singaling).

### Reverse transcription

Total RNA was extracted from cells using Trizol (Invitrogen, Carlsbad, CA). Reverse transcription was performed as preciously described [19] using TaKaRa kit following manufacturer's instruction, in the DNAEngine Peltier Thermal Cycler (BIO-RAD, Richmond, CA). Briefly, RNA and random primers were denatured for 10 min at 70°C; then M-MLV reverse transcriptase, deoxynucleotide triphosphates, RNase inhibitor and reverse transcription buffer were added and incubated for 10 min at 30°C, 60 min at 42°C and 15 min at 70°C.

### Real-time PCR

Primers were synthesized by SBS Genetech (Beijing, China). SYBR Green was used to quantify mRNA levels. All the real-time PCR reagents were purchased from TaKaRa Biotechnology Co. (Dalian, China). PCR reactions were performed as preciously described [19]. Briefly, PCR reactions were performed at the following conditions: 10 seconds at 95°C, then 40 cycles at 95°C for 5 seconds and 65°C for 34 seconds in the ABI Prism 7500 Sequence Detector System (ABI, Foster City, CA). The primers for *MMP-2 *were 5"-CTTCCAAGTCTGGAGCGATGT-3" (foward) and 5"-TACCGTCAAAGGGGTATCCAT-3" (reverse), which amplified a product of 119 bp. The primers for *β-catenin *were 5"-GCTGGGACCTTGCATAACCTT-3" (foward) and 5"-ATTTTCACCAGGGCAGGAATG-3" (reverse), which amplified a product of 86 bp. The primers for *Twist *were 5"-GCCAATCAGCCACTGAAAGG-3" (foward) and 5"-TGTTCTTATAGTTCCTCTGATTGTTACCA-3" (reverse), which amplified a product of 83 bp. *Glyceraldehyde-3-phoshate dehydrogenase (GAPDH) *was used for normalization. The *GAPDH *primers were 5"-CCACCCATGGCAAATTCC-3" (foward) and 5"-GATGGGATTTCCATTGATGACA-3" (reverse), which amplified a product of 71 bp.

### Measurement of reactive oxygen species (ROS)

DCFH-DA fluorescent probes were used to measured the intracellular generation of hydroperoxide (H_2_O_2_) and superoxide anions (O_2_^·-^), respectively, using Reactive Oxygen Species Assay Kit (Beyotime Institute of Biotech, Jiangsu, China), following the manufacture's instruction [20]. Briefly, L9981 cells were incubated with or without BITC or PEITC for 4 h, then reacted with 10 μM of DCFH-DA for 30 min at 37°C. The ROS levels were detected by flow cytometry. The fluorescence was measured at excitation 488 nm and emission 525 nm.

### Measurement of glutathione (GSH)

DTNB were used to measure the intracellular GSH by Total Glutathione Assay Kit (Beyotime Institute of Biotech, Jiangsu, China), following the manufacture's instruction [21]. Briefly, L9981 cells were incubated with or without BITC or PEITC for 3-24 h, cell lysates were prepared, and reacted with assay solution for 5 min at 25°C. The absorbance at A_412 _was measured on a Spectra Max M5 microplate reader (Molecular Devices, Sunnyvale, CA). The GSH concentrations were determined by comparison with standards.

### DNA transfection

Transfection of L9981 cells was carried out using lipofectamine 2000 (Invitrogen, Carlsbad, CA), following the manufacture's instruction. Briefly, L9981 cells were plated in a 24-well plate at 1 × 10^5 ^cell/well. Cells were co-transfected with 400 ng of pNFκB-luc, and 4 ng of pRL-SV40 as an internal control. Cells were rested for 8 h after transfection, then were incubated with or without BITC or PEITC for 18 h. Luciferase assay were performed using the Dual-luciferase Reporter Assay System (Promega) following the manufacture's instruction, on BERTHOLD TriStar LB 941 (BERTHOLD TECHNOLOGIES, Bad Wildbad, Germany)

### Statistical analysis

The data were presented as mean ± standard deviation (S.D.). IC_50 _is the median growth inhibitory concentration value, calculated using GraphPad Prism 5.0 software (La Jolla, CA). Variance analysis between groups was performed by one-way ANOVA and significance of difference between control and treatment groups was analyzed using Dunnett multiple comparison test. The differences with p < 0.05 were considered statistically significant.

## Results

### Effect of isothiocyanates on growth of L9981 cells and NL9980 cells

**Figure 2:**
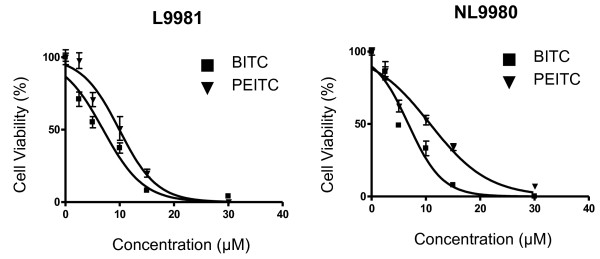
**Effects of BITC and PEITC on growth of highly and low metastatic cells**. L9981 and NL9980 cells were treated with 1 - 40 μM of BITC or PEITC for 48 h, and were collected and counted by a Vi-CELL Cell Viability Analyzer. Values represent the mean ± SD from three independent measurements.

To study lung cancer cell metastasis, a proper metastatic cell model is important. We have established a pair of highly metastatic cell line L9981 and low metastatic cell line NL9980, from a human lung large cell carcinoma cell line (WCQH29801) by the single cell cloning technique [18]. Using this model, we investigate the effect of isothiocyanates on lung cancer cell metastasis. When BITC and PEITC were incubated with low metastatic NL9980 cells, there was a dose-dependent inhibition of cell growth. Both compounds inhibited the growth of NL9980 cells with similar potency: the IC_50 _values were 8.8 ± 0.15 μM for BITC, and 12.2 ± 0.82 μM for PEITC. BITC was more effective than PEITC (Figure 2). BITC and PEITC had a similar effect on the growth of highly metastatic L9981 cells, the IC_50 _values were 5.0 ± 0.22 μM and 9.7 ± 0.39 μM, respectively. Again, BITC was more effective than PEITC. When compared the inhibitory effect of isothiocyanates on highly metastatic cell line L9981 and low metastatic cell line NL9980, we found that isothiocyanates were slightly more potent on highly metastatic cells than low metastatic cells. As this study was to investigate the effect of isothiocyanates on lung cancer cell metastasis potential, the further studies were focused on the highly metastatic cell line L9981. The doses we used were the IC_50 _values of BITC and PEITC.

### Effect of isothiocyanates on migration of highly metastatic L9981 cells

**Figure 3:**
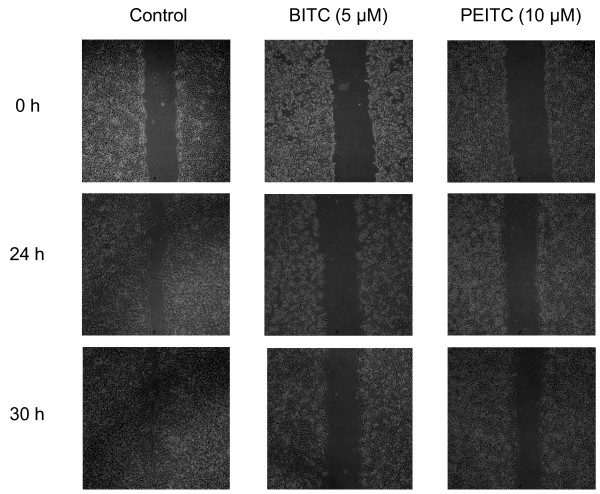
**Effect of BITC and PEITC on L9981 cells migration**. Wound healing assays were performed to assess cell migration. Cells were treated or untreated with 5 μM of BITC or 10 μM of PEITC for 24 and 30 h. Representative photographs of treated and untreated cells are presented (×40 magnification).

**Figure 4:**
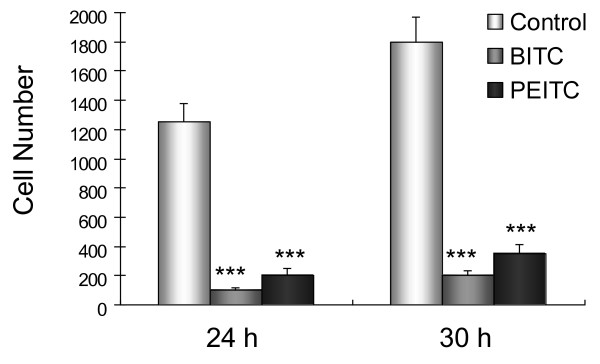
**Effect of BITC and PEITC on L9981 cells migration**. Wound healing assays were performed to assess cell migration. Cells were treated or untreated with 5 μM of BITC or 10 μM of PEITC for 24 and 30 h. Number of cells migrated at 24 and 30 h time point are presented. Values represent the mean ± SD of three independent experiments (*** P < 0.001).

We examined the effect of BITC and PEITC on the migration of L9981 cells by wound healing assay. The doses we used were the IC_50 _values of BITC and PEITC, which did not cause cell death during the experiment. When L9981 cells were incubated with BITC and PEITC, the cellular motility were inhibited in a time-dependent manner. As shown in Figure 3, BITC at 5 μM and PEITC at 10 μM effectively inhibit cell migration after 24 and 30 h incubation, migration levels were reduced to 11.1% and 19.4% of control after 24 h (P < 0.001), respectively; and 8.1% and 16.5% of control after 30 h (P < 0.001), respectively (Figure 4).

### Effect of isothiocyanates on invasion of highly metastatic L9981 cells

**Figure 5:**
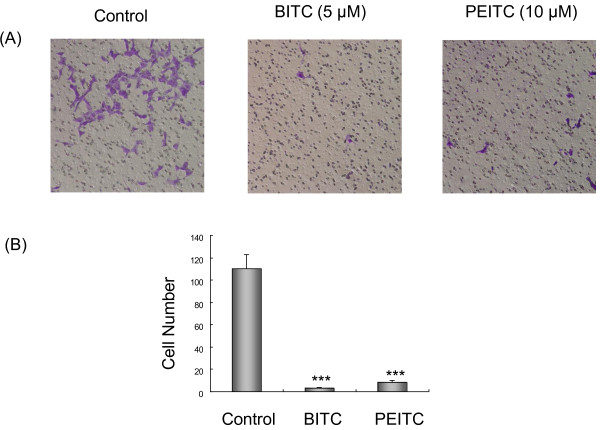
**Effect of BITC and PEITC on L9981 cells invasion**. Invasion Chamber Assays were performed to assess the effect on cell invasion. Cells were treated or untreated with 5 μM of BITC or 10 μM of PEITC for 24 h. (A) Representative photographs of treated and untreated cells are presented (×200 magnification). (B) Number of cells invaded at 24 h time point. Values represent the mean ± SD of three independent experiments (*** P < 0.001).

Invasion is another important step for metastasis. We assessed the inhibitory effect of BITC and PEITC on the ability of L9981 cells to invade a reconstituted extracellular matrix (ECM). BITC and PEITC inhibited cell invasion in a dose-dependent manner. When L9981 cells were grown on Matrigel, a significant reduction in the number of invasive cells was observed when the cells were treated with BITC or PEITC for 24 h, compared to the control. The levels of invasion were reduced to 2.7% and 7.3% of control levels at 5 μM of BEITC and 10 μM of PEITC (P < 0.001), respectively (Figure 5). A significant reduction in invasion was not observed when the cells were treated with lower doses of BITC or PEITC.

### Modulation of metastasis-related genes

**Figure 6:**
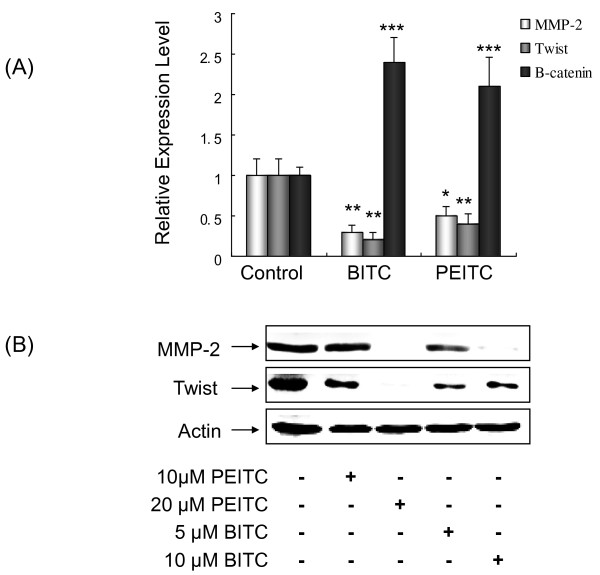
**Effect of BITC and PEITC on metastasis-related gene expression**. (A) L9981 cells were treated with 5 μM of BITC or 10 μM of PEITC for 4 h. *MMP-2*, *Twist *and *β-catenin *mRNA expressions were detected by real-time PCR. (B) L9981 cells were treated with 5 - 20 μM of BITC or PEITC for 24 h. MMP-2 and Twist protein expression were detected by Western blotting analyses (* P < 0.05, ** P < 0.01, *** P < 0.001). Similar results were obtained in three independent experiments.

*MMP-2, Twist *and *β-catenin *play important roles in lung cancer metastasis. *MMP-2 *and *Twist *promote metastasis; whereas *β-catenin *inhibits metastasis. As BITC and PEITC inhibited L9981 cells migration and invasion, we further investigated their effects on these metastasis-related genes. L9981 cells were treated with 5 μM of BITC or 10 μM of PEITC for 4 h, the mRNA expression levels of these three genes were detected by real-time PCR. mRNA expression levels of pro-metastasis gene *MMP-2 *were reduced to 32% and 51% of control by BITC and PEITC (P < 0.05), respectively; mRNA expression levels of pro-metastasis gene *Twist *were reduced to 35% and 43% of control by BITC and PEITC (P < 0.01), respectively. Whereas mRNA expression levels of anti-metastasis gene *β-catenin *were increased. They were increased 2.4- and 2.1-fold by BITC and PEITC (P < 0.001), respectively (Figure 6A). We further detected the protein expression of these genes. Western blotting data demonstrated that both MMP-2 and Twist expression were reduced by BITC and PEITC, in a dose-dependent manner (Figure 6B). These results were consistent with migration and invasion assay results.

### Effect of isothiocyanates on ROS generation

**Figure 7:**
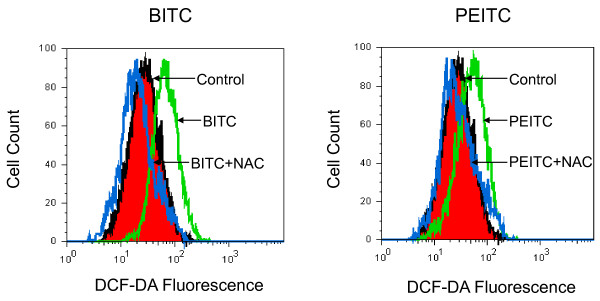
**Effect of BITC and PEITC on ROS generation**. L9981 cells were treated with 5 μM of BITC or 10 μM of PEITC for 4 h, then reacted with DCFH-DA. ROS levels were detected by flow cytometry. For NAC protection, cells were pretreated with NAC (1 mM) for 1 h.

We investigated whether the generation of intracellular ROS is part of the mechanism by which isothiocyanates suppress the metastasis potential of lung cancer L9981 cells. The generation of ROS by isothiocyanates was assessed by using fluorescent probes DCFH-DA by flow cytometry. Treatment with 5 μM of BITC or 10 μM PEITC showed similar effects, resulted in an increase in ROS levels, compared with control (Figure 7). However these were only short term treatments. After a prolonged time, when the Nrf2 targeted genes are expressed, the amount of ROS could decrease. We further investigated the effect of antioxidant NAC on ROS generation. NAC (1 mM) was added to the medium 1 h prior to isothiocyanate treatment, and remained in the medium throughout the experiments. Pretreatment with NAC completely blocked the increased ROS generation induced by both BITC and PEITC.

### Effect of isothiocyanates on intracellular glutathione levels

**Figure 8:**
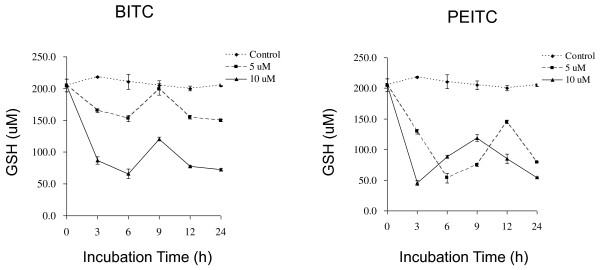
**Effect of BITC and PEITC on intracellular GSH levels**. L9981 cells were treated with 5 or 10 μM of BITC or PEITC for 3 - 24 h, intracellular total GSH levels were detected at indicated time points by microplate reader. Values represent the mean ± SD of three independent experiments.

Glutathione is an intracellular antioxidant, helps protect cells from ROS such as free radicals and peroxides. Whether isothiocyanates exacerbated oxidative stress by causing depletion of intracellular glutathione was investigated. Our data showed both BITC and PEITC decreased total GSH concentration in a dose-dependent manner, but the total GSH concentration in control incubations did not change significantly. When L9981 cells were incubated with 5 or 10 μM of BITC, there was a decrease in total GSH concentration in the initial 3 h of incubation, and continues to decrease till 6 h, but by 12 h had recovered to high level. Thereafter, a further marked decrease occurred until 24 h (Figure 8). When L9981 cells were incubated with 5 or 10 μM of PEITC, total GSH concentration decreased in the 3 to 6 h period. Similar to BITC treatment, they were recovered to high level at 9 or 12 h, respectively. Then declined again and remained at low levels thereafter.

### Effect of isothiocyanates on Akt activation

**Figure 9:**
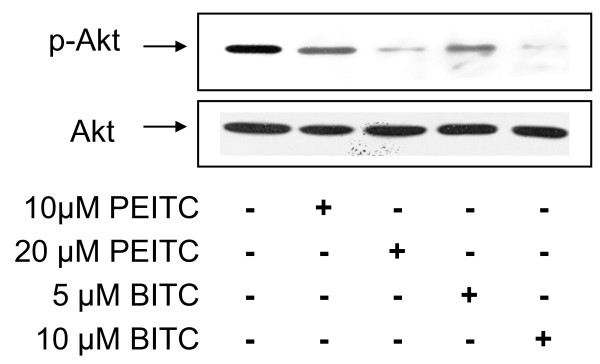
**Effect of BITC and PEITC on Akt activation**. L9981 cells were treated with 5 - 20 μM of BITC or PEITC for 24 h. Cell lysates were prepared, p-Akt and total Akt were detected by Western blotting analyses. Similar results were obtained in three independent experiments.

Akt is an important cell signaling molecule. It blocks apoptosis, and promotes cell survival. Akt has been implicated as a major factor in many types of cancer. To evaluate whether Akt is a target of isothiocyanate on inhibition of lung cancer cell metastasis, we detected the Akt activation by western blotting (Figure 9). Both BITC and PEITC decreased Akt phosphorylation, in a dose-dependent manner. At high concentrations, PEITC (20 μM) and BITC (10 μM) nearly completely blocked Akt phosphorylation; meanwhile, total Akt level remained unchanged.

### Effect of isothiocyanates on NFκB transcriptional activation

**Figure 10:**
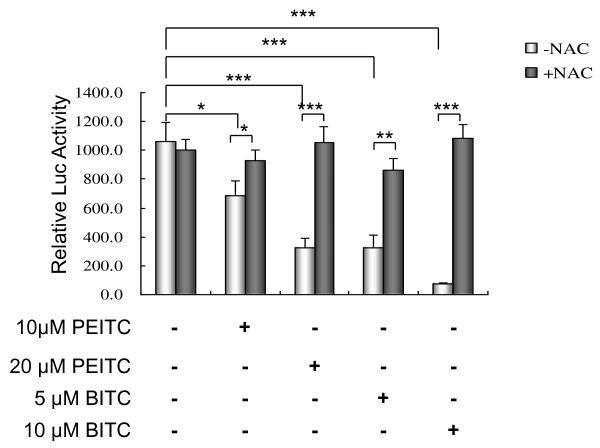
**Effect of BITC and PEITC on NFκB transcriptional activation**. L9981 cells were transfected with pNFκB-luc, and treated with 5 - 20 μM of BITC or PEITC for 18 h. NFκB activation was detected by luciferase reporter assay. For NAC protection, cells were pretreated with NAC (1 mM) for 1 h. Values represent the mean ± SD of three independent experiments (* P < 0.05, ** P < 0.01, *** P < 0.001).

The nuclear factor kappa B (NF-κB) is believed to play an important role in tumor cell growth, proliferation, angiogenesis, invasion, apoptosis and survival. In this study, we investigated the effects of BITC and PEITC on NF-κB transcriptional activation, by luciferase reporter assay. As shown in Figure 10, both BITC and PEITC inhibited the transcriptional activation of NF-κB in a dose-dependent manner. After treatments for 18 h, compared with control, 10 and 20 μM of PEITC significantly inhibited the transcriptional activity of NF-κB to 64.5 and 30.5% of control (P < 0.05), respectively. Similar to PEITC, 5 and 10 μM of BITC significantly inhibited the transcriptional activity of NF-κB to 30.8 and 6.8% of control (P < 0.001), respectively. We further investigated the protective effect of antioxidant NAC. Pretreatment with antioxidant NAC (1 mM) for 1 h significantly attenuated the inhibitory effect of BITC and PEITC on NF-κB transcriptional activation (P < 0.05).

## Discussion

The antiproliferative, antitumour activity of dietary isothiocyanates has been of recent research interest [9]. These compounds have inhibitory effects on several types of cancer cell growth, such as leukemia [22,23], prostate cancer [14], breast cancer [11,17], lung cancer [24], cervical cancer [16], colorectal cancer [15] etc. Our previous studies have demonstrated that allyl isothiocyanate (AITC), PEITC, PETC-Cys and related compounds induce apoptosis in HL60 cells [22,25]. The activation of caspases and JNK are part of the mechanism [23].

Metastasis is the most common cause of death in cancer patients. Therefore, the research and development of novel anti-metastatic drugs is one of the most active fields in cancer research. Recent studies revealed that isothiocyanates have anti-angiogenic and anti-metastatic effects. Isothiocyanates inhibited tumor-specific angiogenesis by down-regulating nitric oxide, TNF-alpha and proinflammatory cytokine production, and by inactivation of Akt [26-28]. Isothiocyanates also suppressed the metastasis potential of human hepatoma cells [29], colon cancer cells [30] and breast cancer cells [31]. This effect is mediated by decreasing the expression of MMPs, pro-inflammatory cytokines, growth factors such as platelet-derived growth factor (PDGF) and vascular endothelial growth factor (VEGF), transcription factor twist; and increasing the expression of tissue inhibitors of matrix metalloproteinase (TIMPs). However, there is no report on the effect of isothiocyanates on lung cancer metastasis. In the present study, we investigated the effect of BITC and PEITC on lung cancer cell metastasis potential, by using a highly metastatic human large cell lung cancer cell line as an *in vitro *model.

We performed wound healing and transwell chamber assays to examine the effect of BITC and PEITC on lung cancer cell metastasis potential, at the concentrations which did not cause cell death during the assays. Our results clearly demonstrate for the first time, that both BITC and PEITC effectively inhibit lung cancer cell migration and invasion *in vitro*. We then detected the effect of isothiocyanates on metastasis-related gene expression. MMPs are a family of zinc-binding endopeptidases that collectively degrade most of the components of extracellular matrix (ECM), and they are necessary for cancer invasion and metastasis [32]. In particular, MMP-2 degrades components of the basement membrane and is strongly implicated in the invasion and metastasis of malignant tumors [32]. Our data showed that BITC and PEITC reduced MMP-2 expression at both mRNA and protein level. Transcription factor Twist is a key regulator of tumor metastasis and an inducer of epithelial-mesenchymal transition. It plays an essential role in metastasis. Twist over-expression correlates with hepatocellular carcinoma metastasis. Suppression of Twist expression in highly metastatic mammary carcinoma cells specifically inhibits its metastatic ability [33,34]. In our study, BITC and PEITC down-regulated Twist expression at both mRNA and protein levels. Another metastasis correlated gene we examined is β-catenin. β-catenin is an epithelial marker, it is necessary for the creation and maintenance of epithelial cell layers. It is down-regulated during lung cancer cell invasion and metastasis [35]. We found that when BITC and PEITC suppressed cell metastasis potential, β-catenin expression was increased. Taken together, these data indicated BITC and PEITC suppressed lung cancer cell metastasis potential by modulating metastasis-related gene expression.

To further explore the underlying mechanism, we investigated the effect of BITC and PEITC on cell survival pathways. Akt/NFκB is a major anti-apoptotic/pro-survival pathway that is frequently hyperactivated in most cancers [36,37]. Akt phosphorylation promotes cell growth and survival by inactivating downstream pro-apoptosis substrates such as Bad, caspases, and activating cell survival substrates such as NFκB. Both clinical analysis and *in vivo *studies showed that Akt plays an important role in cancer cell metastasis [38,39]. NFκB is a transcription factor that is activated by various intra- and extra-cellular stimuli such as cytokines, oxidant-free radicals, ultraviolet irradiation, and bacterial or viral products. It controls the expression of numerous genes involved in immune and inflammatory responses, cell proliferation, oncogenesis, angiogenesis and apoptosis. Inhibition of NFκB activation effectively suppressed tumor cell invasion [40]. More interestingly, recent studies suggested that the activation of Akt/NFκB pathway contribute to the migration of lung cancer cell [41,42]. In this study, we examined the effect of BITC and PEITC on Akt/NFκB pathway. BITC and PEITC inhibited both Akt phosphorylation and NFκB transcriptional activation, in a dose-dependent manner. This suggested that Akt/NFκB pathway is a potential target of BITC and PEITC.

The altered cellular redox status and increased generation of ROS have long been observed in cancer cells, especially the cells in advanced stage tumor, which exhibit multiple genetic alterations and high oxidative stress. This drives us to investigate the effect of isothiocyanates on ROS generation. ROS is generated intracellularly as byproducts of normal aerobic metabolism or as second messengers in various signal transduction pathways or in response to environmental stress. ROS is essential for biological functions. They regulate many signal transduction pathways by directly reacting with and modifying the structure of proteins, transcription factors and genes to modulate their functions. ROS is involved in signalling cell growth and differentiation, regulating the activity of enzymes, mediating inflammation by stimulating cytokine production, and eliminating pathogens and foreign particles [43]. Cancer cells frequently exhibit high oxidative stress. The generation of ROS is part of the mechanism by which most chemotherapeutic agents or ionizing radiation kill tumor cells [44,45]. Recent studies demonstrate that ROS also plays an important role in cell invasion. It regulates cell invasion via MMPs expression, MAPK pathways and NFκB activation [46-48]. In this study, we investigated the role of ROS in isothiocyanate-induced inhibition of lung cancer cell metastasis. Our finding provide evidence of the generation of ROS by BITC and PEITC in lung cancer highly metastatic cells, this is consistent with studies in other type of cancer, such as leukaemia [49], breast cancer [13] and pancreatic cancer [12]. The hypothesis of the increased generation of ROS in response to BITC and PEITC was further supported by the finding that pretreatment with NAC, a general antioxidant, blocked the ROS accumulation. NAC pretreatment also blocked the suppression of NFκB activation, this is in agreement with the finding that ROS-NFκB pathway mediates TGF-beta1-induced cell invasion [48]. It has been described that isothiocynates cause release of Nrf2 from sequestration by Keap1, and its subsequent translocation into the nucleus. Nuclear Nrf2 activates ARE-elements and induces expression of stress-responsive genes [10]. Although for short term treatment the ROS level increases, we expect that after a long term treatment, the ROS level will decrease due to induction of Nrf2 dependent detoxification and antioxidative genes. We suggested that ROS generation may play a role in the inhibitory activity of isothiocyanates on lung cancer metastasis.

GSH is an antioxidant, helps protect cells from ROS such as free radicals and peroxides, it also maintains exogenous antioxidants such as vitamins C and E in their reduced (active) forms. PEITC is known to conjugate with GSH, leading to its exportation and depletion of cellular GSH. Depletion of cellular GSH leads to ROS accumulation. This is thought to be a major mechanism of PEITC-induced ROS stress in cancer cells. Our previous study showed that GSH depletion is involved in leukaemia cell apoptosis induced by PEITC and its cysteine conjugate [50], a current study showed that GSH concentration was decreased by BITC and PEITC. There were recoveries at 9 and 12 h time points, however, this may be due to the stimulated GSH synthesis when cells detected the GSH level was low. After 24 h, GSH concentration decreased again, this is probably because the dysfunction of cellular GSH synthesis system. Some tumor cells have higher GSH level, this is due to the over-expression of Gamma-glutamyl transpeptidase and an interorgan flow of GSH. The higher GSH level promotes metastatic growth [51]. This suggested that GSH may play a role in tumor cell metastasis. Therefore GSH can be a target for metastasis treatment. In supporting this hypothesis, Mena *et al *sensitized B16 melanoma to combination therapy and eliminates metastatic disease by GSH depletion [52]. Our finding that BITC and PEITC decreased GSH level while suppressed tumor cell metastasis potential, also support this hypothesis.

Our results demonstrate that BITC and PEITC inhibited lung cancer highly metastatic L9981cell proliferation, migration and invasion. Metastasis-related genes were modulated and Akt/NFκB pathway was inhibited. Oxidative stress could be part of the mechanism by which isothiocyanates suppressed lung cancer cell metastasis potential.

## Conclusion

Isothiocyanates are well-known chemopreventive agents. Understanding the mechanism of action of these compounds may provide valuable information for their possible application in cancer prevention and therapy. There are already a number of studies that evaluate the effects of isothiocyanates in human subjects [53-55], this could potentially facilitate clinical development of isothiocyanates for cancer therapy.

## Abbreviations

PEITC: phenethyl isothiocyanate; BITC: benzyl isothiocyanate; AITC: allyl isothiocyanate; PITC: phenyl isothiocyanate; DMSO: dimethylsulphoxide; NAC: N-acetyl cysteine; ROS: reactive oxygen species; NF-κB: nuclear factor kappa B; DCFH-DA: 6-carboxy-2,7-dichlorodihydrofluroscein; GSH: glutathione; IC_50_, the median growth inhibitory concentration value; MMP: matrix metalloproteinases

## Competing interests

The authors declare that they have no competing interests.

## Authors' contributions

KX, XW, and QHZ designed the study and wrote the manuscript. XW also performed the signaling pathway experiments. YZ performed cell proliferation experiment, ROS and GSH assays, signaling pathway and transfection experiments. HQY performed the migration, invasion and gene expression experiments. BNL and YL performed cell proliferation experiment. All authors read and approved the manuscript.

## Pre-publication history

The pre-publication history for this paper can be accessed here:

http://www.biomedcentral.com/1471-2407/10/269/prepub
